# Research Trends in Collaborative Drones

**DOI:** 10.3390/s22093321

**Published:** 2022-04-26

**Authors:** Michel Barbeau, Joaquin Garcia-Alfaro, Evangelos Kranakis

**Affiliations:** 1 School of Computer Science, Carleton University, Ottawa, ON K1S 5B6, Canada; barbeau@scs.carleton.ca (M.B.); kranakis@scs.carleton.ca (E.K.); 2 Télécom SudParis, Institut Polytechnique de Paris, 91120 Palaiseau, France

**Keywords:** drone, distributed computing, countering drone threats, target recognition, navigation, risk avoidance, cellular technologies, management of anonymity

## Abstract

The last decade has seen an explosion of interest in drones—introducing new networking technologies, such as 5G wireless connectivity and cloud computing. The resulting advancements in communication capabilities are already expanding the ubiquitous role of drones as primary solution enablers, from search and rescue missions to information gathering and parcel delivery. Their numerous applications encompass all aspects of everyday life. Our focus is on networked and collaborative drones. The available research literature on this topic is vast. No single survey article could do justice to all critical issues. Our goal in this article is not to cover everything and include everybody but rather to offer a personal perspective on a few selected research topics that might lead to fruitful future investigations that could play an essential role in developing drone technologies. The topics we address include distributed computing with drones for the management of anonymity, countering threats posed by drones, target recognition, navigation under uncertainty, risk avoidance, and cellular technologies. Our approach is selective. Every topic includes an explanation of the problem, a discussion of a potential research methodology, and ideas for future research.

## 1. Introduction

Drones have already demonstrated their potential. Their agility allows them to navigate and accomplish tasks in various indoor and outdoor environments. Application examples are the shipping and delivery of essential supplies to remote locations, safe contactless delivery or information gathering for disaster management, geographic mapping of inaccessible terrains, aid in thermal scanning for search and rescue operations, aid farmers for precision land monitoring, law enforcement and border control surveillance, storm tracking, forecasting hurricanes and tornadoes, military surveillance, parcel delivery, and sewer inspection [1,2,3,4,5,6,7].

Drone research is well established [8,9]. Key research areas include drone dynamics, navigation, path planning, and formation control. In this article, rather than cover everything, we offer a personal perspective on a few selected research topics that we envision might lead to fruitful future investigations. Each of the topics that we cover will play an essential role in developing future drone technologies. For every subject, we describe the main challenges, discuss potential approaches and methodology, and develop ideas for further research. More specifically, we explore the six specific problems listed in the sequel.

The first problem deals with collaborative drone applications, in which we assume services that are delegated to cloud services. We provide a representative scenario of distributed computing for the generation, management, and distribution of pseudonyms. The second problem relates to the issue of countering drone threats. Indeed, drones are a perfect example of dual-use technology. They can be used for beneficial as well as malicious purposes. While the adoption of drones simplifies unauthorized video and photo recordings of protected areas, it can also lead to the release of dangerous materials in the proximity of critical infrastructures. Examples include attacks carried out in military missions and flying drones in proximity to airport areas. According to [10], only in 2017, there were more than 300 reported incidents involving small drones. Similar issues are even more popular in the public media [11]. While specific drone applications have countless benefits, others may be a real threat to safety and security. There is a need to develop a science for countering drone threats.

The following three problems we address relate to specific algorithms for collaborative drone applications. First, we explore algorithms for target recognition from the air at a certain altitude. We establish the foundations of a probabilistic framework. Ideas for future work building on this framework are discussed. Second, we establish foundations for drone navigation in GPS-denied environments in the face of decision-making uncertainties. We discuss how these foundations can lead to future research. We complement the previous approach by exploring a formal framework for risk avoidance by flying drones. Within this framework, we introduce ideas for further investigation.

The final problem that we address relates to cellular technologies in networked collaborative drone applications. While early generations of drones required line-of-sight communications with a ground station to maintain control and push data, such as a video stream, the latest drone generations may benefit from the use of Beyond Visual Line of Sight (BVLOS). BVLOS lifts the pilot-drone line-of-sight constraint [12]. It expands the range of operation of drones and types of applications considerably. However, for safe operation, BVLOS requires reliable and real-time command and control. Connections may also serve other purposes, such as data communication and collaboration between drones. Various questions deserve attention, such as the use of cellular technology to network drones, making drones integral parts of the network infrastructure, such as base station-drone or relay, drones pushing information to the cloud, such as location-related signal strength data, or drones pulling information from the cloud, such as flight awareness data. Networking elevates drones’ capabilities to another level.

The outline of this article is as follows. The management of anonymity in the context of distributed computing is explored in Section 2. Countering drone threats is investigated in Section 3. Target recognition, navigation under uncertainty, and risk avoidance problems are covered in Section 4, Section 5 and Section 6. Trends on networked drones using cellular technologies are discussed in detail in Section 7. Section 8 concludes the paper.

## 2. Distributed Computing with Drones

We start with collaborative drone applications in which services are delegated to edge nodes connected to cloud services. This aims at addressing situations in which cooperative drones may not have enough computational resources. They outsource part of their workload to remote resources using the distributed computing paradigm. We provide as a representative scenario the use of cloud services for the generation, management, and distribution of pseudonyms. The use case can be easily adapted to address other similar distributed computing problems.

### 2.1. Problem Description

Network operators may require that drones broadcast messages announcing their identity and location while executing a given mission. Reasons can include identifying and isolating faulty drones, e.g., drones misbehaving due to failures or errors. Privacy issues deriving from the broadcast of unprotected identifiers on a wireless channel have been extensively reported in the literature. This calls for the use of the distributed computing paradigm to delegate the management of pseudonyms while guaranteeing anonymity [13]. Similar privacy-preserving authentication frameworks for Internet-connected vehicles have been proposed. Trends suggest that networked drones may interact with cloud services to generate, distribute, and report results. In such scenarios, edge cloud nodes connected to the cloud service and within the wireless range of collaborative drones assure the mapping of pseudonyms to real identities. These nodes also correlate the collected information and communicate to the cooperative drones new findings, e.g., lists of misbehaving faulty drones.

### 2.2. Approach and Methodology

**Figure 1:**
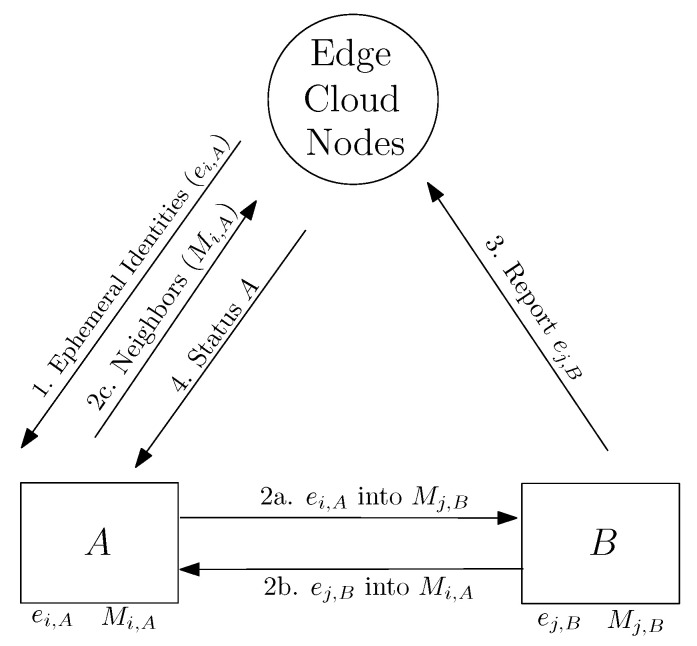
Use of edge cloud nodes to report faulty drones using (anonymous) ephemeral identifiers. Step 1 represents the broadcasting of ephemeral identities to drones *A* and *B*. In steps 2a and 2b, the drone *A*’s data are stored in the memory of drone *B*. In Step 3, drone *B* reports a malfunction to the central service. In Step 4, the central service updates the neighboring drones (e.g., drone *A*, for instance).

As a representative example, we indicate how cloud services can be used to handle the anonymous reporting of faulty drones combining outsourced cloud services with machine-to-machine (short range) communications. Figure 1 offers a centralized approach to generating and broadcasting lists of ephemeral identifiers (e.g., pseudonymous tokens) to collaborative drones using cloud services associated with 5G communications. Later, the drones would exchange those identifiers with other drones hovering nearby, e.g., using machine-to-machine contacts, to report drones showing faulty behavior. The use of either edge or cloud services is assumed for both handling the generation and distribution of ephemeral identifiers as well as to help local drones to report faulty drones to other swarms of collaborative drones.

Step 1 in Figure 1 represents an operation in which a cloud service is broadcasting an ephemeral identity $e_{i,A}$ to drone *A*. This ephemeral identity becomes associated to drone *A* at time *i*. We assume that the cloud service has also generated and broadcasted an ephemeral identity, i.e., $e_{j,B}$ for drone *B* at time *j*.

Notice that the ephemeral identities are time-sensitive. Regularly, they expire, and drones need to request the cloud service to generate and broadcast their new identities to them. We also assume that drones *A* and *B* move around a given terrain in which 5G communications are possible to connect and use those cloud services required to create and distribute new ephemeral identities.

Drones in this scenario are expected to periodically receive and store the ephemeral identities of nearby drones in their local memories. Each drone is expected to receive the identity of other drones via machine-to-machine communications, e.g., using short-range communications such as Bluetooth or IEEE 802.11.

Step 2a in Figure 1 represents an operation in which drone *B* stores the ephemeral identity of drone *A* at time *i* in its local memory. $M_{j,B}$ represents the action of storing those ephemeral identities received by user *B* at time *j*. Similarly, Step 2b represents drone *A* storing the ephemeral identity of drone *B* at time *i* into its local memory. Action $M_{i,A}$ represents the action of storing those ephemeral identities received by drone *A* at time *i*.

Periodically, drones report to a central cloud service the lists of ephemeral identities they received via machine-to-machine communications, which they temporarily stored in their local memories. This is represented in Step 2c of Figure 1, in which drone *A* shares the list $M_{i,A}$ with the central server.

Now, let us assume that drone *B* is reported as faulty at time *j* to get blacklisted in the swarm. This situation is depicted in Figure 1 as Step 3, i.e., reporting the ephemeral identity of drone *B* at time *j* to the central server. The central server that holds all the information collected by all the drones can now warn other drones in the same swarm or other swarms in proximity to drone *B*. This is depicted in Figure 1 as Step 4. The central cloud service sends a new status to drone *A*, since this drone may be affected by the faulty behavior reported for drone *B*.

### 2.3. Future Research

The presented approach requires a dedicated infrastructure, which may not be possible in all drone scenarios and applications. For instance, cloud services and edge nodes to assure the mapping of pseudonyms to real identities may not fit amateur drone applications, i.e., mini-quadcopter drones without an Internet connection. Similar issues have been reported in the anonymity literature related to, e.g., Vehicular Ad-hoc Networks (VANET)s [14]).

In such VANET scenarios, privacy-preserving strategies leverage mix-network ideas that could also be implemented in collaborative drone applications. The use of group schemes [15,16] can be complemented with the help of privacy zones, in which entry and exit from a zone can force the update of pseudonyms. The approach can also be extended to crowd area scenarios [17], in which swap pseudonym strategies [18] and obfuscation techniques can help increase the level of anonymity between groups of drones. The main limitation of those approaches is the necessity of dedicated infrastructure and human-operator assistance.

We envision machine-to-machine communication solutions without the necessity of deploying a cloud infrastructure to access edge computing services. Such future trends can benefit from alternative schemes considered in avionic and maritime contexts. For instance, the authors in [19] propose secure pseudonym schemes for aircraft in which dedicated entities, referred to as trusted registration authorities, oversee assisting aircraft to generate pseudonyms that are evolving. The generation process guarantees irreversible procedures with respect to tracing back a pseudonym to its real identity. Continuous interaction with the operators associated with the trusted authorities may be an important limitation for drone applications. Moreover, data exfiltration from such trusted authorities may reveal the correspondence between pseudonyms and real identities. Future trends can also be inspired by applications in the maritime domain, in which pseudonym strategies have been enforced over marine vessels via traditionally trusted authorities [20]. The main difference is using an infrastructure-free approach, although a persistent connection to the registration authority is still required. Methods such as those proposed in [21] for the secure and anonymous broadcast of identifiers assume a secure communication channel among mobile vehicles.

**Figure 2:**
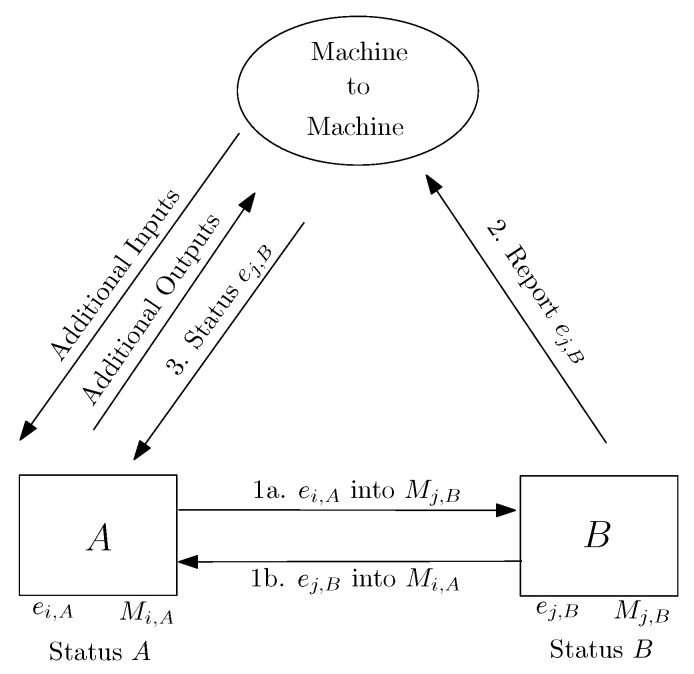
Distributed architecture to report faulty drones using anonymous ephemeral identifiers. In contrast to the approach depicted in Figure 1, drones exchange lists of ephemeral identities and locally update their status without the necessity of central intermediary services. The main differences with respect to the centralized version are in Steps 2 and 3, which are conducted now in a machine-to-machine mode.

Inspired by the previous ideas, we envision some alternative methodologies to conduct the anonymous collection and dissemination of information reported in this section, but limiting as much as possible the dependence of drones to cloud infrastructure services as those referred to in Figure 1. A decentralized version of the previous approach is depicted in Figure 2. In this new approach, drones create the list of ephemeral identities by themselves. This way, now both drones *A* and *B* manage their lists of ephemeral identities, broadcasting and expiring them at regular time intervals. When drones such as *B* are reported as faulty, they can be sensed and reported to other drones in the neighborhood by using the list of ephemeral identities previously exchanged when they were still behaving correctly.

In contrast to the approach illustrated in Figure 1, drones can now request and obtain lists of ephemeral identities and locally check whether or not their neighbors are potentially faulty. Remote cloud and edge services can be used as a redundant resource to store, forward, and broadcast lists of potentially defective drones identified by their ephemeral identities. In some mission-specific applications, a delegation of distributed processing to edge nodes and cloud services leads to assumptions and issues, which are not always possible to solve—for example, in situations where the deployment of dedicated infrastructure and centralization is not possible. At the same time, the use of distributed computing with collaborative drones may lead to more complex scenarios when managing, e.g., fault diagnosis, which is a promising future research field in collaborative drone scenarios with well-established foundations from cooperative multi-agent systems [22,23]. We consider that further work remains to address such additional scenarios.

## 3. Countering Drone Threats

Drones can be used in illicit activities. Several cases have been documented lately. In Saudi Arabia, terrorists used drones to attack oil pumping stations [24]. A Turkish company has developed an autonomous suicide drone [25]. Drones flying close to airports, business centers, and elsewhere in densely populated areas pose a direct danger to critical infrastructure and humans. There are numerous incidents involving rogue drones violating airspace. Drones have also been used to smuggle drugs into the walls of jails [10]. These incidents call for technology to protect infrastructures and populations from harmful activities perpetrated leveraging drones.

### 3.1. Problem Description

The problem is threefold:

1. *Spotting and Following.* When do drones enter a given airspace? Are they friends or adversaries? What tasks are drones specially fitted for? Are they armed? Loaded? What is their kind? How many rotors do they have? Are they small? Micro? Are they equipped with infrared cameras? Are drones autonomous or under the command and control of a human pilot? What are the whereabouts of spotted drones?
2. *Intention determination.* What is the drones’ mission? Are they looking around infrastructures? Searching? Are they preparing for a strike? What is their target? Are they taking pictures? Shooting videos? What strategies or algorithms are they following to achieve their mission?
3. *Riposte.* How to respond to the presence of adversarial drones? What are the effective countermeasures?

The swarm dimension, drones acting in a collaborative group to achieve their goal, and the increasing degree of elaborateness of drones compound the problem. The environmental conditions are also challenging, since drones are equipped with sensors of various types and have onboard computing resources that enable embedding advanced algorithms and artificial intelligence, rendering them reactive and adaptable.

### 3.2. Approach and Methodology

The sub-problems can be approached either passively or actively. Table 1 lists the countering drone threat tasks together with various ideas that exist and sometimes have been investigated in the research community. Conventional radar technology is not practical for spotting and following drones, especially for micro-drones flying at slow speed and low altitudes and for areas such as urban environments. Luckily, spotting and following can be achieved passively using audio, video, and radio frequency observations [26,27,28]. The use of artificial intelligence and Machine Learning (ML) for solving problems related to countering drone threats has grown considerably. Agents learn and exploit what they have learned to spot and follow drones. Al-Sa et al. have created a Data Base (DB) of drone Radio Frequency (RF) signals. Together with the use of neural networks, the database can be used to detect drone presence, their type, and kind of flight [29]. Wit et al. use micro-Doppler signatures for drone rotary-wing determination, i.e., helicopter, bi-rotor, quadcopter, hexacopter, or octocopter [30]. Research has also been completed to determine the load carried by a drone. Using micro-Doppler signatures, Fioranelli developed an approach to determine whether a drone carries a load or not [31]. Traboulsi and Barbeau further investigated this question and developed a method to determine the weight of the payload of a drone [32]. Schumann et al. [33] use deep learning to build classifiers to discriminate drones from birds. The approaches considered have limited success due to the weaknesses of the classifiers to adversarial learning attacks, as demonstrated by Shamir et al. [34] and Eykholt et al. [35].

On the intention determination front, drone maneuvers recognition has also been investigated, such as the work of Bartak and Vemlelova using Reinforcement Learning (RL) [36]. Using telemetry data, a Supervised Learning (SL) model to predict the formation of a drone swarm aims has been proposed by Traboulsi and Barbeau [37].

Ideas for riposte in response to detecting malicious drone behavior have been quite limited. Attempts to jam the signals of autonomous drones may not be applicable. Shooting flying drones may have their risk.

### 3.3. Future Research

In summary, attention has been given mainly to drone spotting and following. Intention determination and riposte have received little if any attention. More research is needed on all aspects of countering drone threats. Table 1 highlights the lack or absence of solutions to several sub-problems related to the countering of drone threats.

## 4. Target Recognition

In this section, we focus on ways to recognize physical targets using one or more drones, possibly either cloud connected or wirelessly with each other, and which are traveling at a certain speed either away from or toward a (moving) target.

### 4.1. Problem Description

Drones are being used to explore terrestrial regions to discover specific or sought after features and characteristics, such as carrying out human or animal counts, diagnosing specific environmental situations, recognizing a given landmark or sign, or searching for a particular mammal, cf. [38,39]. Additional technical studies also include [40,41,42].

Tasks with low bandwidth requirements can be performed offline by participating ground stations interacting with drones using a cellular network. However, appropriate network bandwidth may not be available. It is more likely that a drone (or swarm of drones) would need to explore the environment, interpret the data collected, and make decisions based on its own online diagnosis. In this section, we ask the question:

How can we accurately detect a (possibly) mobile target from a distance?

We consider only altitude-based detection (see [43,44,45,46]). Considerations could also be extended to speed-based detection (e.g., speed of data sampling) or a combination of altitude and speed.

### 4.2. Approach and Methodology

**Figure 3:**
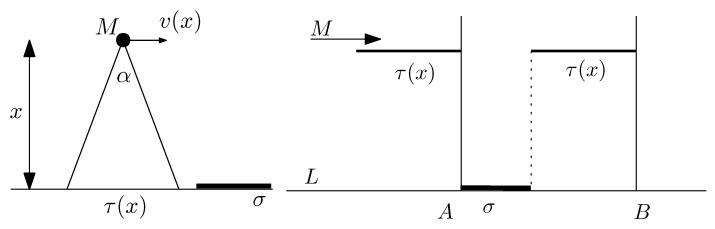
Drone *M* is flying over a line segment *L* of length *ℓ* and a certain object of length σ. At height *x*, the drone flies with speed v(x) which depends on the altitude *x*. The picture in the righthand side indicates that the drone’s camera has a sliding window effect of width τ(x) within which it can detect an object of length σ.

Consider a drone flying over a certain highway represented as a line segment *L* of length ℓ=|L|. During the flight, the drone retains a given altitude *x*. The speed v(x) of the drone may be decided by prevailing regulations on the drone’s flying territory and is a function of *x* (see Figure 3, left picture).

The drone’s camera has a field of view (or visibility angle) α. When flying at height *x*, the drone’s visibility angle α subtends a subsegment of the line of length τ(x); this is the portion of the line *ℓ* on which the drone’s camera can detect objects. Since the field of view of the camera is α and the drone is flying at altitude *x*, we must have that τ(x)=2xtan(α/2) degrees.

First, we determine the target object density as a fraction of the total length *ℓ* of the segment *L*. As the drone is moving, it is detecting objects located on the line segment. The drone’s camera at altitude *x* has a sliding window of width τ(x). This is moving with speed v(x), thus yielding a time of possible observation equal to τ(x)/v(x).

The following analysis is inspired from [38,40]. An object of length σ is placed on the line *L*. As indicated in Figure 3 (right picture), assuming it is moving left to right, the drone starts observing the object at its leftmost endpoint *A*. It stops observing when at the point *B*, thus yielding a total distance of observation equal to σ+τ(x). The total duration of observation is equal to $\frac{\sigma +\tau (x)}{v(x)}$. It follows that the drone is observing the target object a fraction f(x), where

(1) $$f\left(x\right)=\frac{\left(\sigma +\tau (x))/v(x\right)}{\left(\ell -\tau (x))/v(x\right)}=\frac{\sigma +\tau (x)}{\ell -\tau (x)},$$

of its total flying time over the segment of length *ℓ*, assuming that the rightmost point of the target object is at a distance at most τ(x) from the rightmost point of the segment of length *ℓ*. In a way, the righthand side of Equation (1) represents the target object density.

Next, we look at distributions for the probability that the object is detected by the drone. Using distance sampling statistics, one could use the exponential distribution

(2) $$p\left(x\right)=\frac{1}{\ln(1+1/k)}\cdot \frac{e^{-x}}{k+e^{-x}},$$

as the probability that a drone flying at altitude x≥0 detects the target object when its camera is directed toward it.

The parameter *k* appearing in Equation (2) is chosen so as to indicate the probability that an autonomous ground vehicle detects the target when traveling at ground level (altitude x=0), e.g., $p\left(0\right)=\frac{1}{k\ln(1+1/k)}$. The term $\frac{1}{\ln(1+1/k)}$ is the normalizing coefficient and is derived as follows. Observe that $\int \frac{e^{-x}}{e^{-x}+k}dx=x-\ln\left(ke^{x}+1\right)$ and for x≥0, this yields the formula

$$\int_{0}^{\infty }\frac{e^{-x}}{e^{-x}+k}dx=\ln\left(1+\frac{1}{k}\right)$$

Note that the (exponential) probability distribution above is one of several potential choices; others may be considered depending on the situation.

Using the target object density calculations in the previous subsection, we conclude that the probability $P_{\sigma }\left(x\right)$ that the drone detects a target object of length σ is equal to

$$\begin{array}{cc} P_{\sigma }\left(x\right) & :=p\left(x\right)\cdot f\left(x\right)=\frac{1}{\ln(1+k)}\cdot \frac{e^{-x}}{k+e^{-x}}\cdot \frac{\sigma +\tau (x)}{\ell -\tau (x)}, \end{array}$$

where f(x) is the fraction of its total flying time that the drone will be observing a fixed object of length σ over the segment of length *ℓ*.

### 4.3. Future Research

There are several directions for additional research. In case of a searching swarm consisting of *n* drones acting independently of each other, the probability that the target object is detected by at least one of the drones is easily seen to be equal to $1-{\left(1-P_{\sigma }\left(x\right)\right)}^{n}$. One could also consider more general settings by employing p(v,h) as the error probability of image interpretation, namely the probability that the image is interpreted wrongly given that the drone is moving with speed *v* and is at altitude *h* from the target. It should be noted that a similar analysis could be applied if the object itself is mobile. Moreover, one could also explore machine learning techniques to improve target recognition for swarms of cooperating drones.

## 5. Navigating under Uncertainty

This section considers drone navigation under conditions that may cause decision uncertainty in path selection. These may affect the signal’s quality as received or processed by the drone. To this end, we take into account recognition and advice errors.

### 5.1. Problem Description

When drones are navigating an unknown environment, they may have to find a flight path from a source to a destination in a GPS-denied environment using only clues obtained from landmarks visited. In particular, when hovering over an area, they can acquire data through their camera(s) and other sensors, which may be visual, acoustic, etc. Data collected are being used to interpret landmarks. A priori, drones may be given clues and specific characteristics about landmarks that they can use throughout their route discovery. For example, they may be seeking a green door or a tall building. Our main question here is the following:

How can drones navigate through an environment of landmarks without using GPS information?

### 5.2. Approach and Methodology

In the sequel, we outline a technique notivated from the work of [47] and first employed in [48,49] in order to navigate such a terrain.

Consider a terrain with available landmarks, e.g., road signs, monuments, or even other terrestrial indicators. The landmarks encountered may provide information whose interpretation by the drone may be prone to various types of errors. One can distinguish two types of errors, namely, recognition and advice.

- Recognition errors may be due to misinterpretation of sensed data or confusion of objects. For example, a drone has found a green door which in fact is not a door but rather a window leading to an incorrectly recognized object. We assume that for some real number p∈[0,1], the value *p* is the probability that a drone performs recognition erroneously and 1−p that it is correct.
- Advice errors could be about landmarks because the information they provide is either not up to date or even outright wrong. For example, upon finding a landmark, a drone is advised to traverse a certain distance within the terrain in direction north where it will find the next landmark, say a restaurant, but this information is wrong because the restaurant is no longer there. Again, we may assume that for some real number q∈[0,1], the value *q* is the probability that the advice provided to a drone about a landmark is erroneously interpreted and 1−q that it is correctly interpreted.

The next step concerns mathematical assumptions of the model above. To facilitate calculations, one may assume that recognition errors are independent and identically distributed and advice errors are also independent and identically distributed. In other words, the drones are acting independently of each other. In addition, the outcome of the recognition process is random with a probability of success that depends on the parameter *p*. A similar observation applies to the advice process. We can use this methodology to our advantage so as to improve the recognition and advice mechanisms for swarms of drones.

**Figure 4:**
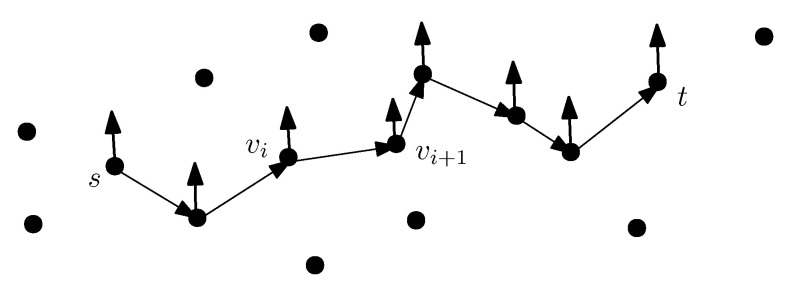
Flight path from source *s* to destination *t*. Edge $\left(v_{i},v_{i+1}\right)$ is an intermediate segment connecting landmarks $v_{i}$ and $v_{i+1}$. The vertical (thicker) arrows represent the landmarks encountered by the drones.

There is an underlying graph determined by the terrain of landmarks. Landmarks are vertices of a graph G=(V,E) whose edges are discovered online by the recognition and/or advice process. In a typical setting, we start with a source node *s* and end with a target node *t*. The drones are seeking a flight path connecting m+1 vertices $s:=v_{0},v_{1},\ldots ,v_{i},v_{i+1},\ldots ,v_{m}:=t$, see Figure 4.

A possible flight path, denoted as *P*, consists of a number of vertices $v_{0}:=s,v_{1},\ldots ,v_{i},$$v_{i+1},\ldots ,v_{m}:=t$ from *s* to *t*. An edge $\left\{v_{i},v_{i+1}\right\}$ corresponds to a segment of the flight path *P*. It is said to be correctly traversed if and only if the advice provided about the landmark associated with vertex $v_{i}$ is valid and correctly interpreted and the landmark associated with vertex $v_{i+1}$ is correctly recognized. For i=0,…,m−1, the flight path *P* is correctly traversed if and only if each of its segments defined by edges $\left\{v_{i},v_{i+1}\right\}$ are correctly traversed.

At the start, a drone is given a flight plan. The flight plan defines the flight path *P*. For each vertex $v_{i}$, i=0,…,m−1, the flight plan comprises advice for searching for the next landmark, such as directional data. For each vertex $v_{i+1}$, the flight plan contains recognition data, such as landmark characteristics. A flight plan is correctly performed solely if every single segment is correctly traversed.

The next important step is for the swarm of drones to fuse information collected so to reach an optimal decision. The basic method is to employ error amplification. Namely, we amplify errors by employing the majority rule and using statistical sampling in order to make decisions on how to navigate the environment (e.g., choose a direction along the ones recommended, accept a certain advice but discard others, and in general improve recognition, etc). Thus, at each landmark being explored, the drones first collect information and make individual decisions on how to interpret the information. Second, they exchange their individual interpretations and decide based on the majority rule by communicating the common decision. Additional details of this can be found in [48,49]. Such an approach turns out to be useful and may lead to improved navigation that may supplement existing navigation techniques.

### 5.3. Future Research

The methodology proposed offers potential for additional research and testing more sophisticated majority strategies based on probability distributions that are realistic in that they are sensitive, for example to geographic location, proximity to base station, and/or landmarks and available energy of drones. Additionally, one could make use of a database (DB) of landmarks and employ machine learning approaches on drone swarms that could improve the overall decision-making process. For example, contextual bandits could be a natural framework for routing and decision making in such a terrestrial environment, cf. [50] for further details.

## 6. Risk and Obstacle Avoidance

This section considers approaches to risk and obstacle avoidance when drones are traveling through a possibly unknown environment. The drones themselves may be equipped with sensors to analyze collected data locally or in the cloud.

### 6.1. Problem Description

When a drone is traversing a given terrain, it is exposed to risks arising from the spatial density of a particular factor being taken into account, e.g., concentration of chemicals, disturbing weather phenomena and/or patterns, environmental pollution, population density, and even the viral load (for a given virus) in a constrained space. Our main question is the following:

How can a drone navigate a risky terrain so as to minimize the impact of hazardous factors?

### 6.2. Approach and Methodology

**Figure 5:**
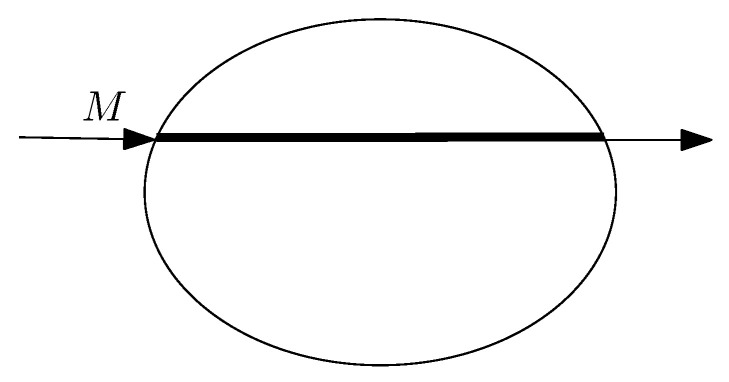
A drone *M* is traversing a straight line trajectory. Its exposure during the trajectory may be measured by the length of the path inside the risky region.

**Figure 6:**
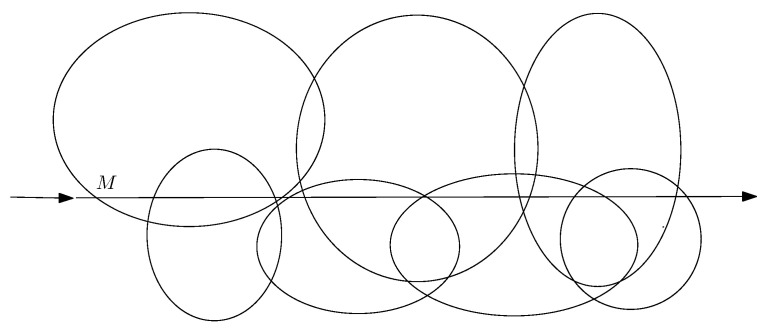
A drone *M* is traversing a straight lime trajectory. Its exposure during the trajectory is measured by the sum of the exposures from each of the regions (depicted as ovals) within its range.

There are many ways to measure the risk when a given region is traversed by the drone. For example, when the source of the risk is not known, it could be measured by the length of the portion of the path traversed by the drone that is inside the given region (see Figure 5), which we also refer to as the exposure. In general, the hazardous region will be enclosed by the perimeter of a complicated domain (represented as oval in Figure 5). However, if the source of the risk is also known (something we refer to as hotspot), then sometimes, it may be possible to measure the risk from either the distance from the hotspot or an area covered by the drone during the traversal (e.g., see Figure 6.) This is of course an idealized scenario, but it can be useful for making computational estimates on the complexity of the problem.

More generally, consider regions in the plane representing risky areas, which were initiated from various sources (which may or may not be known) of pollution, viral infection, population density, etc. As depicted in Figure 6, a drone *M* is traversing a straight-line trajectory in a given terrain. The goal of the drone is to navigate the terrain so as to find a path that minimizes its risk of exposure. In many instances, one might be able to measure this quantity precisely.

**Figure 7:**
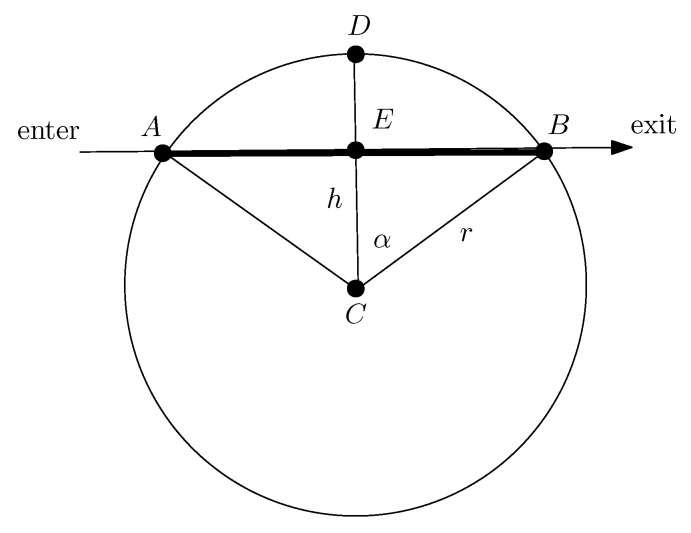
A drone *M* is traversing a straight line trajectory from *A* (entrance) to *B* (exit) at distance *h* from the point *C*. Its path inside the circle is depicted by the thick segment AB.

Measurements can be done in various ways. For example, associated to each region, the authors of [51] consider only disks which are generated by hotspots located at the respective centers of the disks. The critical parameter is a distance r>0 that represents how far the hotspots can reach. The value of *r* depends on the hotspot’s intensity to cause harm, so that the bigger the *r*, the higher the degree of harm that the hotspot can cause. Moreover, beyond *r*, no harm is possible. A drone in proximity to the hotspot may get harmed depending on its distance from it. The amount and intensity of harm depends on the distance between the drone and the hotspot (see [51] for additional details). More specifically, the metric introduced in [51] is defined by the area of the circular sector delimited by the points A,E,B,D; it is easy to see that this satisfies $Area\left(AEBD\right)=\arccos\left(\frac{h}{r}\right)\cdot r^{2}-h\cdot \sqrt{r^{2}-h^{2}}$ (see Figure 7).

For the metric of harm proposed here, one may consider the length of the portion of the trajectory of the mobile inside the disk. For this metric, we observe the following. Assume a drone is traversing a straight line trajectory at distance *h* from a hotspot (e.g., an infected station) with critical distance *r*, where r≥h≥0. The total amount H(r,h) of harm that the drone *M* incurs for its entire trajectory within the hotspot’s range is equal to

(3) $$H\left(r,h\right)=2r\sqrt{1-h^{2}/r^{2}}$$

(see Figure 7 where a drone *M* is traversing a straight lime trajectory at distance *h* from the source *C* entering at *A* and exiting at *B*). Similar estimates may also be possible to derive for other types of regions. There are various kinds of hotspots, for example, for the case of an infection, the function H(·,·) may be the well-known viral load which is studied in epidemics, but in general, it does not have to be limited to this.

More generally, if a drone traverses a path *P* consisting of k+1 vertices $u_{0},u_{1},\ldots ,u_{k}$ with respective sensitivity distances $r_{0},r_{1},\ldots ,r_{k}$, then the total harm accumulated will be equal to

(4) $$\begin{array}{c} \sum_{i=0}^{k}H\left(r_{i},h_{i}\right), \end{array}$$

where $h_{i}$ is the distance of the drone from node $u_{i}$. The summands in Formula (4) as given by Equation (3) are for all the hotspots encountered. Moreover, a drone may be within more than one region at the same time and hence under the influence of more than one hotspot. More generally, different hotspots may cause different types of harm $H_{i}\left(\cdot ,\cdot \right)$, for i=0,…,k. We can develop navigation strategies using this metric that are similar to those developed in [51]. Similar obstacle avoidance navigation algorithms can also be found in [52,53].

### 6.3. Future Research

In our discussion and analysis, we proposed a simple additive model for measuring the viral load accumulated thus leading to a technique for risk avoidance. Additional metrics for complex regions (e.g., polygonal, etc.) could also be explored using algorithmic and optimization techniques inspired from computational geometry. An interesting question would be to give algorithms to determine (or even prevent taking) a path given that a certain threshold cannot be exceeded. For example, if the critical value of harm is *T*, one might assume that a harm occurs if

(5) $$\begin{array}{c} \sum_{i=0}^{k}H\left(r,h_{i}\right)\geq T, \end{array}$$

i.e., the total amount of harm is at least *T*, a threshold value that may well depend on risk and safety considerations as well as sensitivity of the measuring equipment. The models proposed and studied so far are in 2D space; another important case would be to look at more realistic models in 3D space. It would also be interesting to explore other metrics as well as incorporate techniques from Bandit routing, cf. [50], and study machine learning approaches to improve risk avoidance and maintain a safe routing path.

## 7. Networked Drones Using Cellular Technologies

The classical pilot-to-drone connection is assured using Visual Line of Sight (VLOS) communications. A direct link is used between a pilot and a drone. The pilot–drone distance is limited by the capability of maintaining eye contact with a drone, such as 500 m. To go far off VLOS, it has been proposed that cellular network infrastructures be used for pilot-to-drone command and control and more. This concept is called BVLOS communications. Pilots maintain communications with drones across arbitrarily long distances over cellular networks. BVLOS enables the piloting of drones outside the line of sight of pilots. Drone traveling distances become limited solely by cellular network coverage. Drones may be guided in complex structures involving buildings, electric poles, and wiring. Because communications are maintained, pilots can always intervene if something goes wrong. Applications of BVLOS include inspection of infrastructures, such as pipelines, and delivery of parcels, food, and medical supplies in urban or rural areas. BVLOS communications are expected to be one of the important applications of upcoming generations of cellular networks. This realization of this concept enables drone applications requiring wide geographical area coverage.

### 7.1. Problem Description

Research issues that stem from BVLOS include:

1. Low latency connections for drone command-and-control.
2. Interference mitigation due to altitudes of drones.
3. Cooperative drone group communications.

### 7.2. Approach and Methodology

The BVLOS concept has been explored with 4G cellular networks [12]. The 4G cellular networks made possible multimedia applications such as music and video streaming. The latency of 4G networks, in the order of 50 to 200 ms, does not address the real-time needs for drone command and control, which are in the order of a few milliseconds. Furthermore, 4G cellular networks have been designed for serving ground User Equipment (UE). In contrast, flying drones operate at altitudes. It has been observed that 4G cellular communications of flying drones are prone to high interference levels [12]. The resulting low Signal to Interference Ratio (SINR) values are causing degradation and loss of connectivity [2].

Low network latency is a requirement for safe drone command and control. The 5G networks are aiming at network latency in the order of a few milliseconds. The 5G technology being deployed addresses the needs of drone communications. BVLOS is a use case of 5G networks [54,55,56]. BVLOS communications require reliable connections between pilots and drones for aspects such as command and control, telemetry and first-person camera video [57,58]. The 5G network BVLOS communications build upon the Ultra Reliable Low Latency Communication (URLLC) use case [59]. It aims at a latency of a few milliseconds. Another goal is reliability, that is, less than a $10^{-5}$ packet drop rate and less than $10^{-9}$ bit error rate. Pilots can safely operate drones unhindered within these parameters by network traffic load and latency.

### 7.3. Future Research

Drone communications are different from traditional cellular communications due to the tight closed-loop requirements of uplink and downlink communications and the mixed traffic types communicated, such as command and control signals, sensor measurements, and bandwidth-hungry high-definition video and liDAR. Future research should ideally be conducted in the 5G network environment, which aims to overcome the drawbacks of 4G networks concerning the support of drone communications. The 5G networks make drones capable of operating across long non-line-of-sight distances. The wide 5G bandwidth enables more considerable data traffic. The BVLOS concept paves the way for new applications. For example, drones can communicate with each other and ground stations to combine their efforts to achieve a search and rescue mission. The 5G BVLOS communications with drones have their challenges, including propagation channel modeling, 3D trajectory planning, energy-efficient design, and network and radio resource allocation.

An exciting idea is the use of drones to physically carry core network elements [60,61,62,63,64,65]. They can play the role of relay or Base Station (BS). They can be untethered or tethered. The battery’s capacity limits the time of operation of an untethered drone, such as one hour. A tethered drone is powered and linked by a cable. Fly time is not limited by battery capacity. It can last for days. The wired link can provide wider bandwidth than a wireless link. However, being attached to the ground, the range of operation of a drone is limited to a particular zone, typically a truncated hemisphere. Benefits of flying relays or BSs include easy relocation and installation concerning ground relays and BSs. Their lifetime is, of course, limited, especially for untethered BS, which are dependent on the energy reserve of their battery. Research challenges include air-to-ground and air-to-air channel modeling and optimal placement of flying relays or BS. Past research has investigated the quality of ground user connections served by a flying BS and achieving the best possible data rates using machine learning approaches to determine the best location for flying relays.

A challenge in the BVLOS connected drone use cases is ensuring drone connectivity and Quality of Service (QoS) support throughout a drone mission. Ensuring predictable coverage and low communication delays is a non-trivial task due to the phenomenon of scattered cell associations where different altitudes result in varying patterns of received signal power [66]. Classical drone navigation strategies tend to focus on reducing the travel time and energy consumption of drones. However, the optimal navigation path does not likely provide the uplink and downlink QoS requirements of a drone or may unnecessarily consume a large amount of network resources. Minimizing travel time and energy usage while maximizing connectivity and reducing cellular network resources are conflicting requirements that need novel algorithms and approaches to enable widespread connected drone use-case adoption. Another challenge within this domain is drone re-connectivity upon connection disruption. Even with connectivity-aware path planning, a drone may often lose connectivity due to propagation errors or changes in network deployment and increased load. To mitigate this, novel algorithms that can re-route the drone to alternative routes with the required QoS connectivity are needed.

## 8. Conclusions

Collaborative drones use new networking technologies, such as 5G networks and cloud computing. The goal is to expand traditional applications and their horizon from search and rescue to more ambitious scenarios encompassing new aspects of everyday life. The available research literature on the topic is vast. No single survey article could ever cover all the relevant issues. We have presented a personal collection of research themes covering a few selected areas that we consider worth pursuing in future research. We chose a few research trends on networked, collaborative drones, highlighting target recognition, navigation, risk avoidance, use of cellular technologies, anonymity, and countering drone threats. We introduced each topic by including the problem definition, discussion of a potential research approach, and ideas for future research. Each topic represents just one promising solution w.r.t. the applications domains reported in our work. For example, the use of distributed computing with collaborative drones presents clear advantages for the management of communications in a decentralized manner, for instance, to deal with anonymity issues in situations where the deployment of dedicated infrastructure and centralization is not possible. At the same time, the use of distributed computing with collaborative drones may lead to more complex scenarios when managing, e.g., fault diagnosis in cooperative tasks. Further work remains to be done to address such issues.

## Figures and Tables

**Table 1:** Tackling the countering drone threats. The first column lists the tasks. The second column provides passive solutions based solely on observations. The third column indicates active solutions, acting on threatening drone behavior.

	Passive	Active
*Spotting and following*	Audio, video and RF	Autonomy rev. Turing test
	observations and ML	
	RF signal DB	
	Micro-Doppler signatures	
*Intention determination*	Maneuvers recognition w. RL	
	Formation prediction w. SL	
*Riposte*		RF jamming
		Counterattack drones

## Data Availability

Not applicable.
